# Double trouble: a patient with both HLA-B27 anterior uveitis and HLA-A29 birdshot chorioretinitis

**DOI:** 10.1186/s12348-014-0028-6

**Published:** 2014-11-26

**Authors:** Zeina Haddad, Ashvini Reddy

**Affiliations:** Department of Ophthalmology, University of Virginia, 1300 Jefferson Park Avenue, Charlottesville, 22908 VA USA

**Keywords:** Birdshot chorioretinitis, Ankylosing spondyloarthropathy, Seronegative spondyloarthropathy, Panuveitis, Cystoid macular edema

## Abstract

**Background:**

Birdshot chorioretinitis (BSCR) is a rare ocular inflammatory disorder associated with HLA-A29 and characterized by bilateral choroidal lesions, vitritis, macular edema, and retinal vasculitis. Ocular inflammation associated with HLA-B27 is typically a recurrent, unilateral, acute anterior uveitis (AAU) that is frequently associated with ankylosing spondylitis (AS). To date, there are no reports of patients with both HLA-A29-positive BSCR and HLA-B27 AAU/AS in the English literature.

**Findings:**

A 50-year-old man with a history of bilateral anterior uveitis, vitritis, retinal vasculitis, and cream-colored depigmented oval choroidal lesions was found to be HLA-A29 and HLA-B27 positive. His lumbar spine and sacroiliac joint films revealed fusion of the spine, known as `bamboo spine' compatible with the diagnosis of ankylosing spondyloarthropathy. He had chronic ocular inflammation that was difficult to control with systemic steroids and immunomodulatory agents.

**Conclusions:**

This is the only report of a patient with both HLA-A29-positive BSCR and HLA-B27-positive AS and associated anterior uveitis. The severity of his disease suggests that patients who test positive for both HLA-A29 and HLA-B27 carry a poor visual prognosis. Prompt diagnosis and treatment with local or systemic corticosteroids or steroid-sparing agents may control the disease.

**Electronic supplementary material:**

The online version of this article (doi:10.1186/s12348-014-0028-6) contains supplementary material, which is available to authorized users.

## Findings

### Introduction

Birdshot chorioretinitis (BSCR) is a rare ocular disorder characterized by bilateral choroidal lesions and chronic intraocular inflammation without anterior segment complications. A minimum of three discrete, round or oval, cream-colored foci of depigmentation is required for diagnosis. The lesions are most often one quarter to one half optic disc diameter in size and clustered around the disc, nearly always with involvement of the inferior and nasal peripapillary area. Exclusion criteria include keratic precipitates and posterior synechiae, which may form as a consequence of independent HLA-B27-related anterior uveitis [1].

### Case description

In 2002, a 38-year-old male sought care for flashes and floaters, blurred vision, intermittent eye redness, and photophobia OU. Initial best corrected visual acuity was 20/20 OU with bilateral peripheral field constriction, vitritis, and choroidal lesions. At that time, he was diagnosed with bilateral panuveitis with cystoid macular edema (CME) and started on 40 mg oral prednisone daily and topical steroid and nonsteroidal anti-inflammatory drops. A laboratory workup included complete blood count, erythrocyte sedimentation rate, antinuclear antibodies, rapid plasma reagin, fluorescent treponemal antibody absorption test, angiotensin converting enzyme, chest X-ray, and toxoplasmosis serology - all of which were within normal limits. In 2004, his visual acuity deteriorated to 20/60 OU. A diagnostic vitrectomy was performed in the left eye and was negative for malignancy and infection. Vision failed to improve, despite bilateral cataract surgery, due to persistent bilateral vitritis, episodes of anterior uveitis, CME, and epiretinal membranes. He was treated only with steroid and nonsteroidal anti-inflammatory drops over this time period.

**Figure 1:**
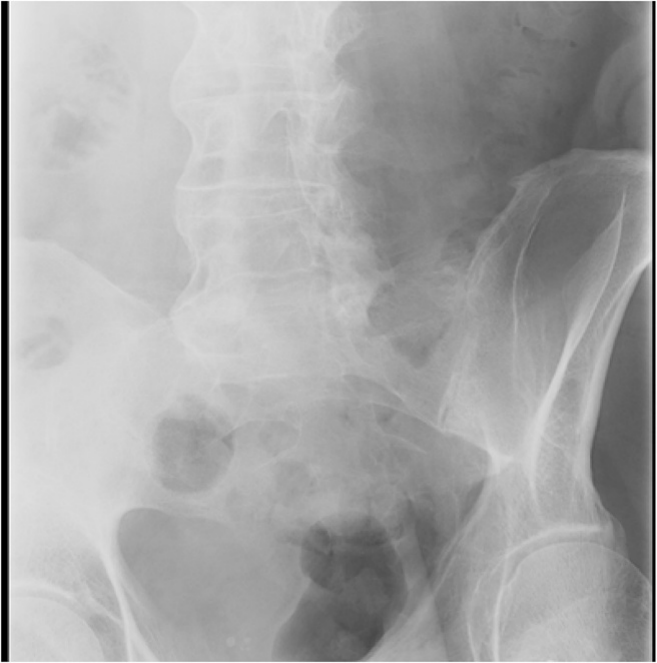
**X-rays of sacroiliac joints and lumbar spine.** Show partial fusion of the bilateral sacroiliac joints, lumbar spine syndesmophytes, and fusion of the posterior spinous processes known as `bamboo spine.

In 2008, he was seen in follow-up and noted to have persistent ocular inflammation. His vision was 20/50 OU. His slit lamp examination was notable for mild vitritis bilaterally and 1+ haze. He had no cells or flare in his anterior chamber. Fundus examination was remarkable for bilateral optic nerve pallor, cream-colored depigmented oval choroidal lesions most numerous nasal to the optic disc, retinal vessel attenuation, epiretinal membranes, and moderate CME with attenuation of foveal contour. A diagnosis of BSCR was suspected. A repeat laboratory workup was ordered along with HLA typing. Complete blood count, erythrocyte sedimentation rate, C reactive protein, antinuclear antibodies, rheumatoid factor, rapid plasma reagin, fluorescent treponemal antibody absorption test, angiotensin converting enzyme, chest X-ray, tuberculosis skin test, and Lyme, toxocara, and toxoplasmosis serologies were within normal limits. His HLA typing was positive for HLA-A29. He was started on prednisone 60 mg daily and referred to a rheumatologist, who noted cervical spine abnormalities with abnormal gait and posture. X-rays showed partial ankylosis of the bilateral sacroiliac joints and lumbar spine syndesmophytes (Figure 1). HLA-B27 typing was subsequently positive, and he was diagnosed with BSCR and ankylosing spondylitis (AS). He was started on methotrexate 20 mg weekly, but this failed to control his ocular inflammation. He was switched to mycophenolate mofetil 1,000 mg daily, which was later increased to 1,500 mg daily as prednisone was being tapered.

**Figure 2:**
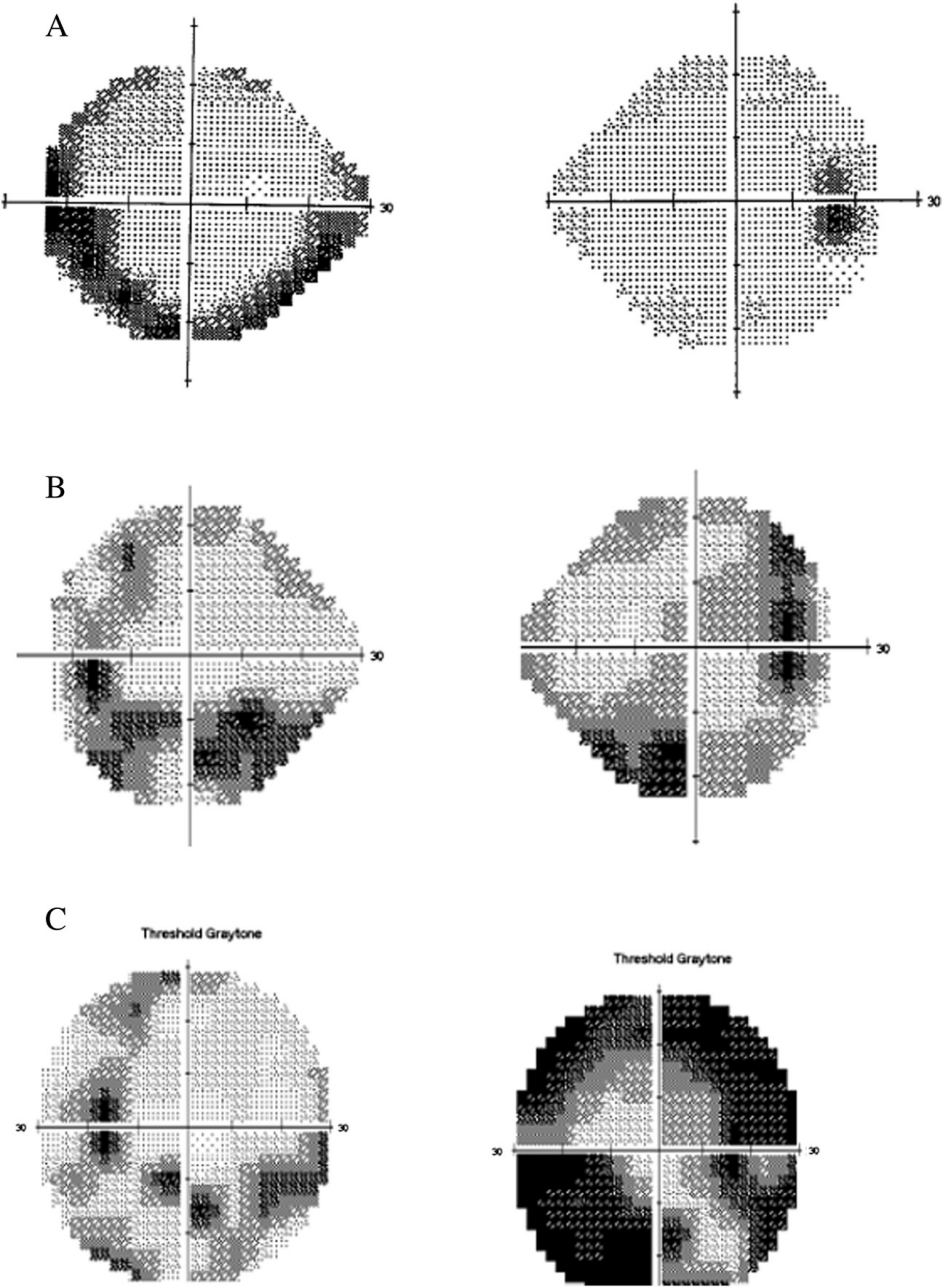
**A-24-2 Humphrey visual fields done in 2002.** Showing left inferior arcuate defect, enlarged blind spot, and few scattered desaturation points inferiorly in the right eye. **B**-24-2 Humphrey visual fields done in 2010 showing progressive worsening of visual field defect with bilateral inferior and superior arcuate defect more pronounced in the left eye with desaturation points around fixation in the right eye. **C**-30-2 Humphrey visual fields done in 2014 showing further progression of visual field defect with peripheral constriction and enlarged blind spot more pronounced in the right eye.

**Figure 3:**
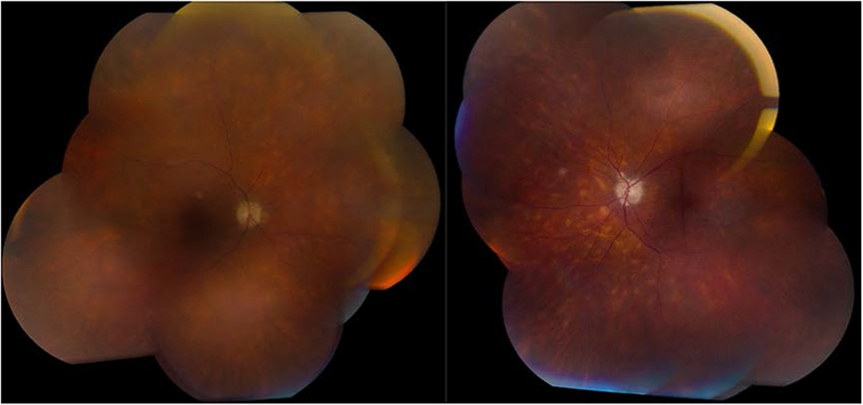
**Montage fundus photos of the right and left eye.** Showing cream-colored oval choroidal lesions scattered throughout the fundus.

**Figure 4:**
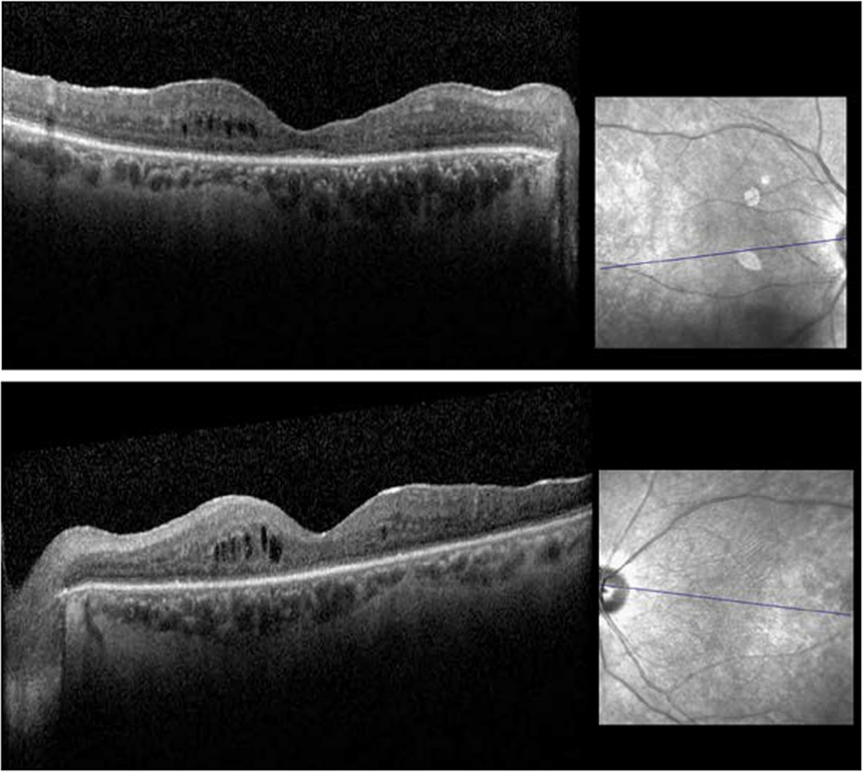
**Spectral domain optical coherence tomography of the right and left eye.** Showing epiretinal membranes, cystoid macular edema, and atrophic retina.

**Figure 5:**
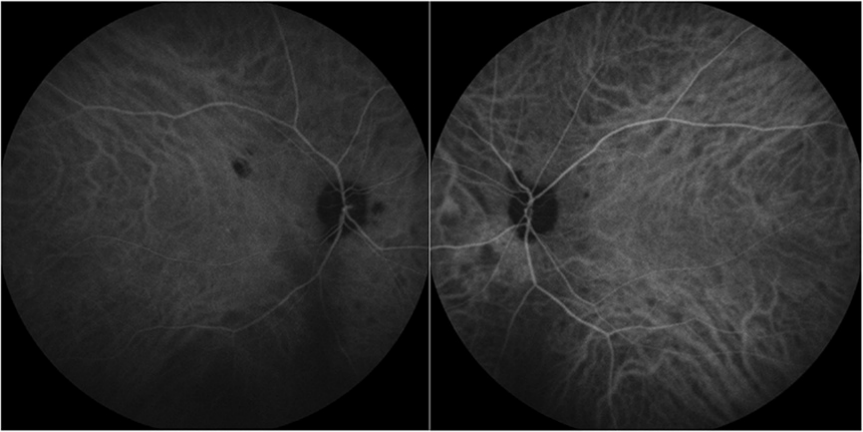
**Indocyanine angiography revealed hypofluorescent areas corresponding to choroidal lesions.**

On his most recent examination in 2014, vision was 20/200 OD and 20/80 OS. His slit lamp examination was remarkable for rare cells in the anterior chamber OS and moderate vitritis with 2+ haze OU. There had been progressive worsening of visual fields (Figure 2). Fundus examination revealed stable bilateral choroidal lesions, optic nerve pallor, retinal vessel attenuation, epiretinal membranes, and mild cystic edema with attenuation of foveal contour (Figures 3 and 4). The patient reported an allergy to fluorescein dye. Indocyanine green angiography revealed hypofluorescent dark spots corresponding to birdshot lesions (Figure 5). His anti-inflammatory and rheumatic therapeutic regimen consisted of mycophenolate mofetil 500 mg TID for ocular inflammation and Voltaren and Vicodin for pain related to his AS. Steroid implant or a second steroid-sparing agent was recommended given his progressive disease. However, the patient declined any additional therapy and preferred close monitoring.

The patient provided consent for the report to be published.

## Discussion

In 1975, Ryan and Maumenee [2] used the descriptive term birdshot retinochoroidopathy to define a rare ocular inflammatory disease characterized by bilateral cream-colored ovoid spots densest around the optic nerve and nasally.

Classically, eyes with BSCR are quiet with no ciliary injection or anterior chamber inflammation. Common posterior segment findings include hyperpermeable capillaries with CME, narrowing of retinal arterioles, perivascular hemorrhages, vessel tortuosity, and optic disc swelling. HLA typing is a valuable diagnostic tool but not a requirement for diagnosis; it has a sensitivity of 96% and a specificity of 93% [3].

The required characteristics for the diagnosis of BSCR according to Levinson [1] are bilateral disease, the presence of at least three peripapillary `birdshot lesions inferior or nasal to the optic disc in one eye, low-grade anterior segment intraocular inflammation (defined as ≤1+ cells) and a low-grade vitreous inflammation (defined as ≤2+ vitreous haze). Birdshot lesions are defined as cream-colored, irregular or elongated, choroidal lesions with indistinct borders, the long axis of which is radial to the optic disc. The ocular inflammatory activity was assessed according to the Standardization of Uveitis Nomenclature Working Group. Supportive findings for the diagnosis of BSCR were: HLA-A29 positivity, retinal vasculitis, and CME. Exclusion criteria for the diagnosis of BSCR were the presence of keratic precipitates and posterior synechiae and the presence of infectious, neoplastic, or other inflammatory diseases that can cause multifocal choroidal lesions [1]. Exacerbations and remissions characterize the course of the disease. Loss of visual acuity is due to CME, macular epiretinal membrane formation, macular hole, subretinal neovascular membrane, macular scar, and cataract [4].

Our patient with AS-related acute anterior uveitis (AAU) was given a diagnosis of BSCR based on the clinical exam and had HLA typing confirming HLA-A29 and HLA-B27 status. Over 90% of patients with AS are HLA-B27 positive, whereas only 6% to 8% of the general population is HLA-B27 positive. The primary ocular manifestation of AS is recurrent nongranulomatous acute anterior uveitis, which our patient reported. Acute anterior uveitis occurs in 25% to 40% of patients with AS [5].

Priem and Oosterhuis reviewed 62 patients with BSCR. In half of the patients followed for 5 years or more, visual acuity was maintained at 20/60 or better [4]. More recent studies suggest that central visual acuity can be preserved long term in patients with BSCR. Tomkins-Netzer et al. found that 88% (*n* = 81) and 97% (*n* = 89) of the eyes maintained best corrected visual acuity and did not progress to permanent visual loss (<20/50) or severe visual loss (<20/200), respectively over 10 years [6]. Our patient had uncontrolled ocular inflammation despite being on systemic steroids and immunomodulatory agents. Vision deteriorated to 20/200 OD and 20/80 OS with associated nerve pallor, epiretinal membranes, macular edema, and retinal atrophy. The severity of his disease suggests that patients who test positive for both HLA-A29 and HLA-B27 carry a poor visual prognosis, though delay to diagnosis and patient deferral of additional treatment are also contributing factors.

BSCR is generally considered to be an isolated ocular disorder. Few reports in the literature suggest a possible association with systemic illnesses including essential hypertension, cerebrovascular accidents, hearing loss, and cutaneous immune-mediated conditions such as vitiligo and psoriasis [4],[7]-[10].

To our knowledge, this is the only reported case of a patient with both HLA-A29-positive BSCR and HLA-B27-positive AS and associated anterior uveitis. Given the severity of his disease, we suspect that patients with this association develop inflammation that is difficult to control and have poorer visual prognosis than patients with BSCR alone. Prompt diagnosis and treatment with local or systemic corticosteroids or steroid-sparing agents may be of benefit.

## Authors' information

ZH is a third-year ophthalmology resident at the University of Virginia and had completed a two-year vitreoretinal fellowship at the University of Virginia. AR is a uveitis and a medical retina specialist at the University of Virginia.

## Authors’ original submitted files for images

Below are the links to the authors’ original submitted files for images. Authors’ original file for figure 1 Authors’ original file for figure 2 Authors’ original file for figure 3 Authors’ original file for figure 4 Authors’ original file for figure 5 Authors’ original file for figure 6 Authors’ original file for figure 7

## Abbreviations

AAU: acute anterior uveitis
AS: ankylosing spondylitis
BSCR: birdshot chorioretinitis
CME: cystoid macular edema
