# Supplementary figures and images for: Double trouble: a patient with both HLA-B27 anterior uveitis and HLA-A29 birdshot chorioretinitis
Source: J Ophthalmic Inflamm Infect. 2014 Nov 26;4:28. doi: 10.1186/s12348-014-0028-6 (PMC4883984; doi:10.1186/s12348-014-0028-6)

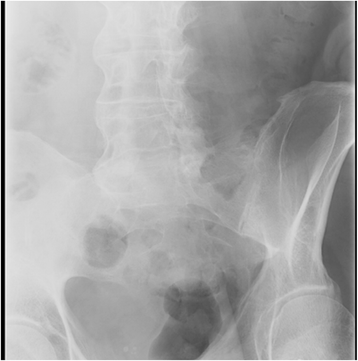

Supplement: Supplementary file 1 — Authors’ original file for figure 1 [file 12348_2014_28_MOESM1_ESM.gif]

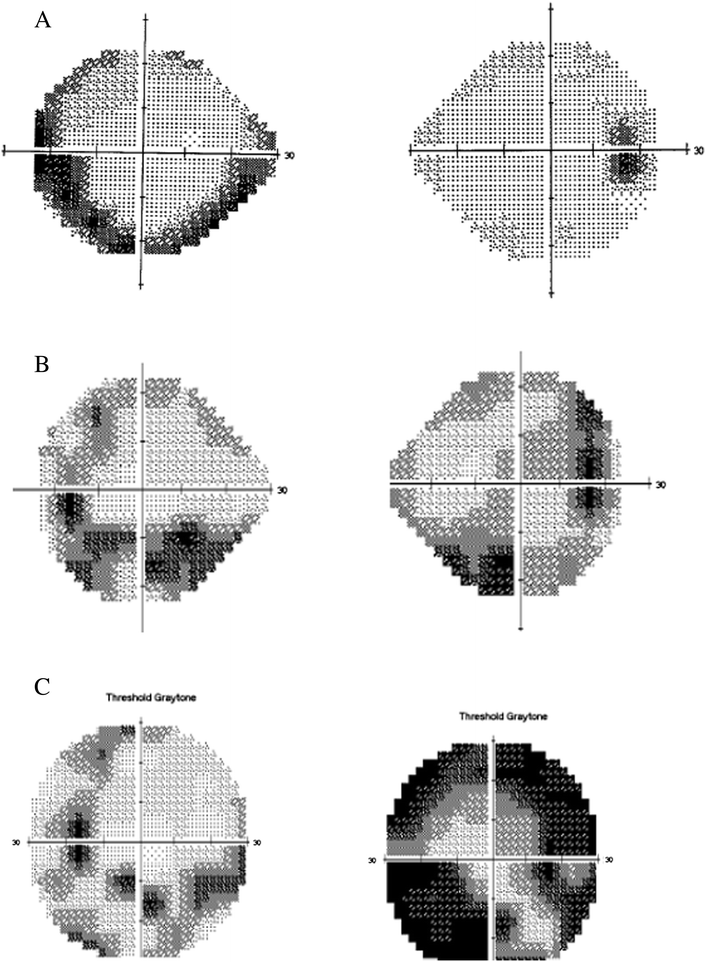

Supplement: Supplementary file 2 — Authors’ original file for figure 2 [file 12348_2014_28_MOESM2_ESM.gif]

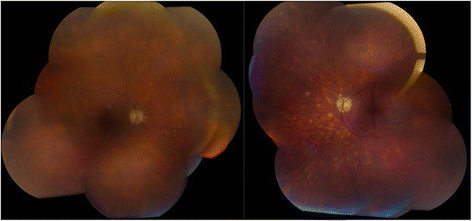

Supplement: Supplementary file 3 — Authors’ original file for figure 3 [file 12348_2014_28_MOESM3_ESM.gif]

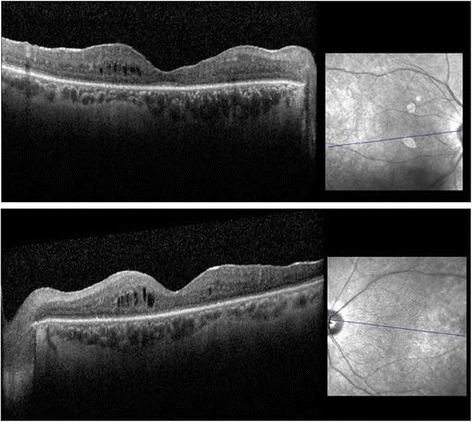

Supplement: Supplementary file 4 — Authors’ original file for figure 4 [file 12348_2014_28_MOESM4_ESM.gif]

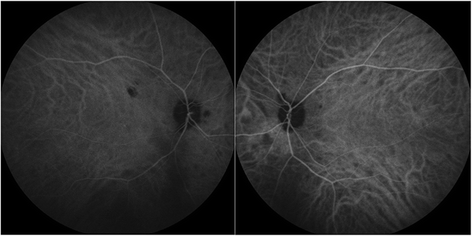

Supplement: Supplementary file 5 — Authors’ original file for figure 5 [file 12348_2014_28_MOESM5_ESM.gif]

Figure 4:


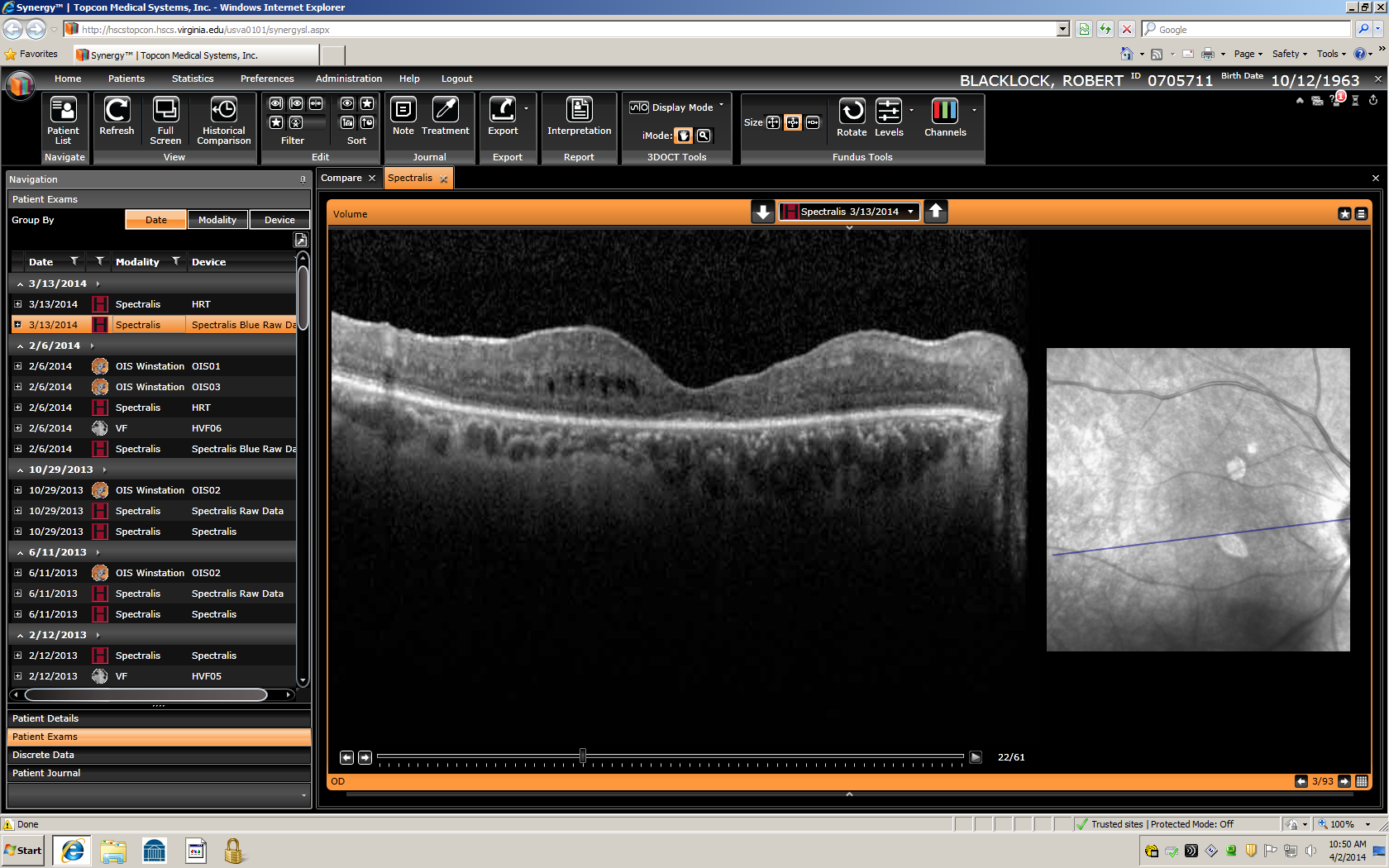


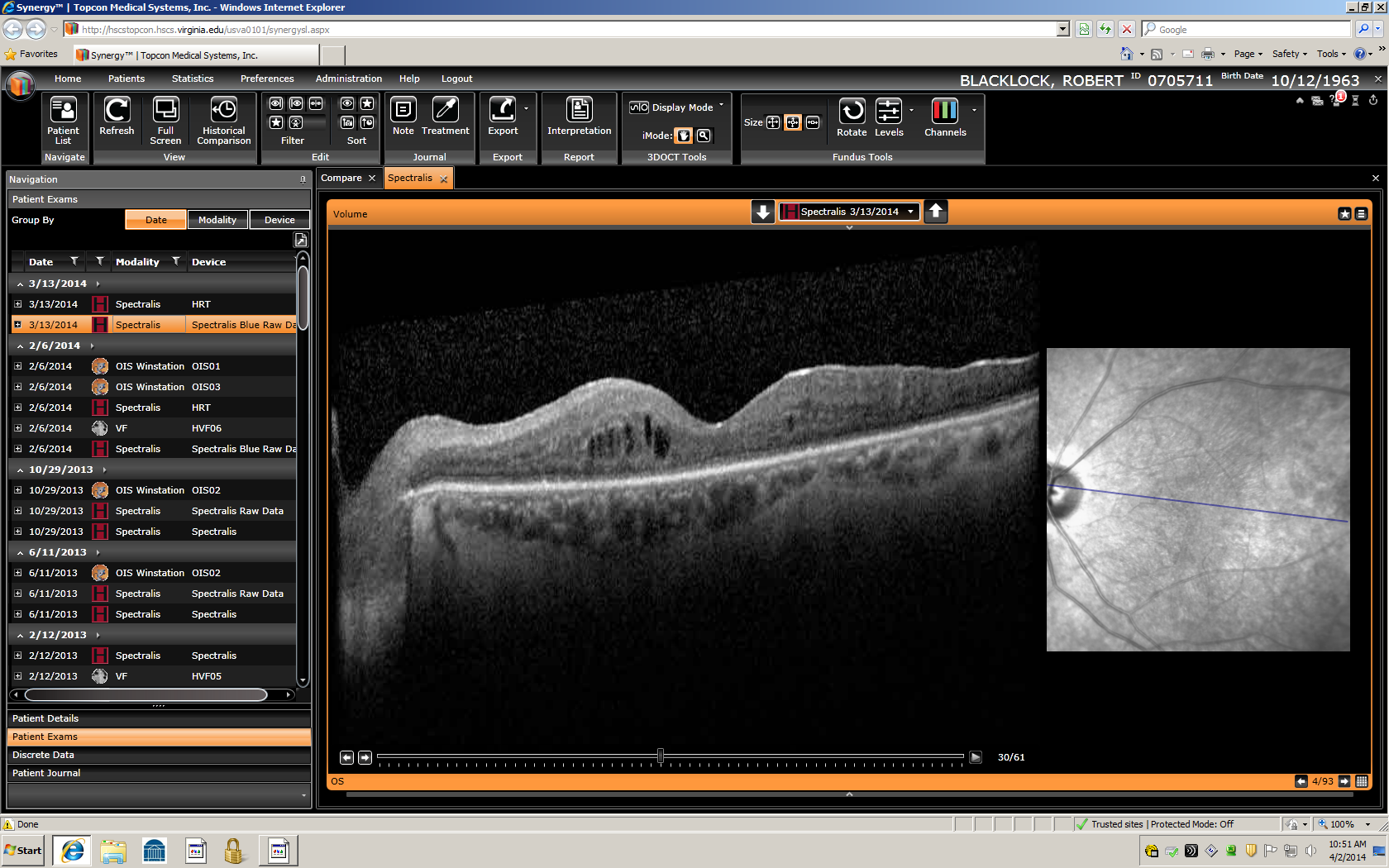

Supplement: Supplementary file 6 — Authors’ original file for figure 6 [file 12348_2014_28_MOESM6_ESM.docx]

Figure 5:


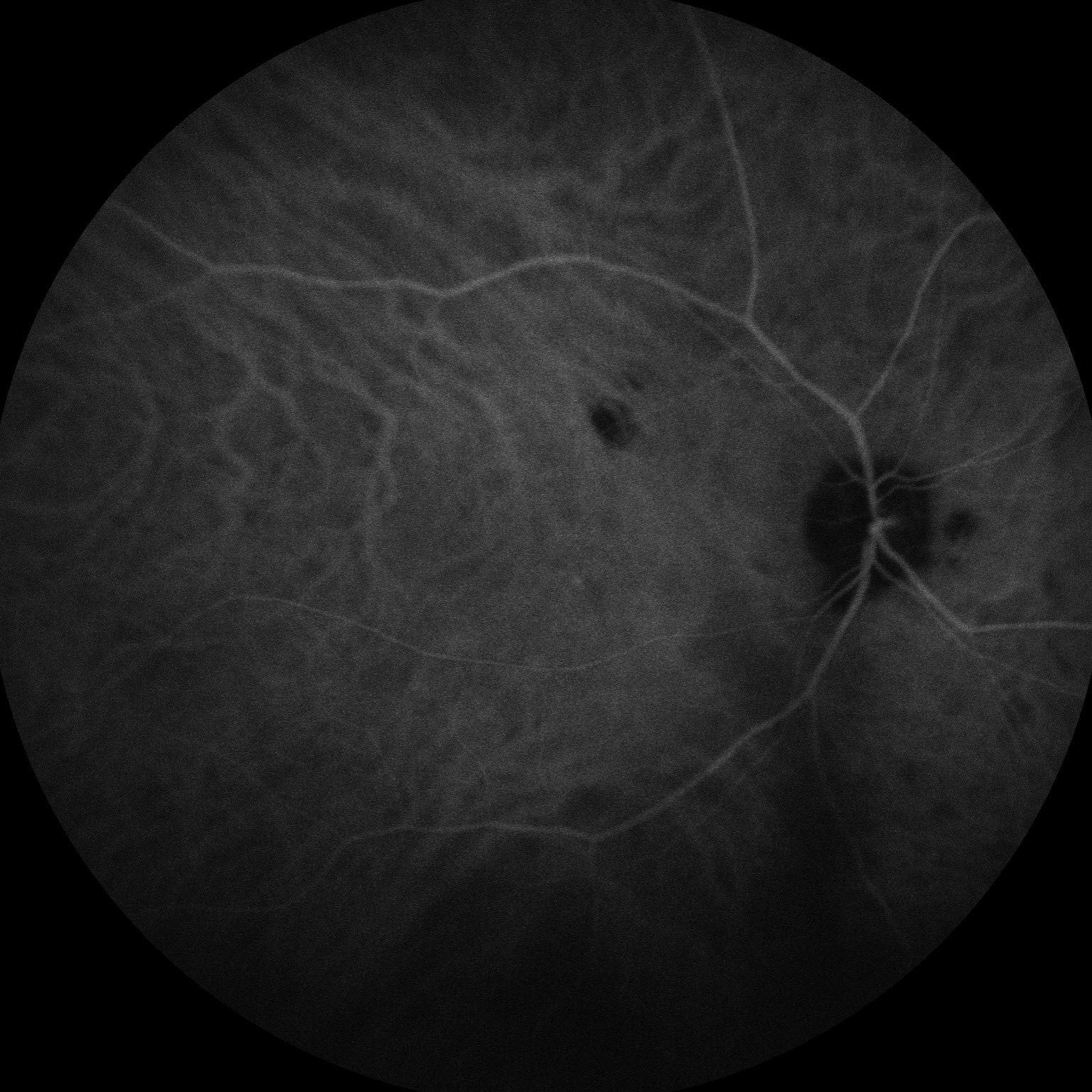

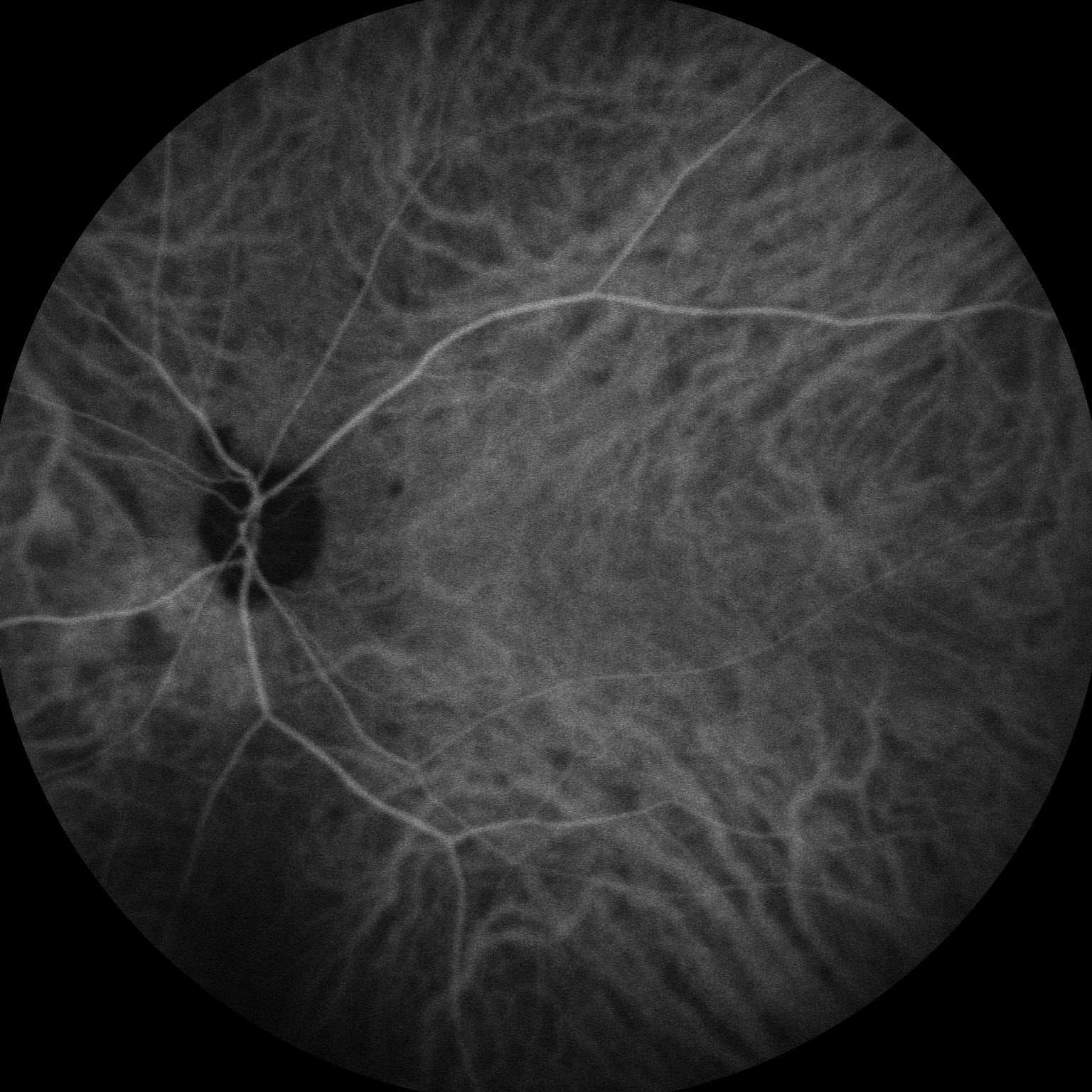

Supplement: Supplementary file 7 — Authors’ original file for figure 7 [file 12348_2014_28_MOESM7_ESM.docx]
